# Induction of pro-inflammatory response of the placental trophoblast by *Plasmodium falciparum* infected erythrocytes and TNF

**DOI:** 10.1186/1475-2875-12-421

**Published:** 2013-11-15

**Authors:** Ana María Vásquez, Cesar Segura, Silvia Blair

**Affiliations:** 1 Grupo Malaria, Facultad de Medicina, Universidad de Antioquia UdeA, Calle 70 No. 52-21, Medellín, Colombia

**Keywords:** Placental malaria, Syncytiotrophoblast, Cytoadherence, Inflammatory activation, ICAM-1

## Abstract

**Background:**

*Plasmodium falciparum* placental malaria is characterized by the sequestration of infected erythrocytes (IEs) in the placental intervillous space via adherence to chondroitin sulphate A (CSA), production of inflammatory molecules, and leukocytes infiltration. Previous reports suggest that the syncytiotrophoblast (ST) immunologically responds to IEs contact. This study explores the inflammatory response induced in BeWo cells by adherence of IEs and TNFstimulation.

**Methods:**

A non-syncitialized BeWo cells (trophoblast model) were used to evaluate its response to CSA-adherents IEs (FCB1csa, FCB2csa, FCR3csa, 3D7csa) and TNF stimulation. Expression of membrane ICAM-1 (mICAM-1) receptor in BeWo cells was quantified by flow cytometry and the IL-8, IL-6 and soluble ICAM-1 (sICAM-1) concentrations were quantified by enzyme-linked immunosorbentassay (ELISA) in BeWo stimulated supernatants.

**Results:**

BeWo cells stimulated with TNF and CSA-adherents IEs of FCB1csa and 3D7csa (strains with higher adhesion) increase the expression of ICAM-1 on the surface of cells and the secretion of immune factors IL-8, IL-6 and sICAM-1. This inflammatory response appears to be related to the level of adherence of IEs because less adherent strains do not induce significant changes.

**Conclusions:**

It was found that BeWo cells responds to CSA-IEs and to TNF favouring a placental pro-inflammatory environment, evidenced by increases in the expression of membrane mICAM-1 and release of soluble ICAM-1, as well as the IL-8 and IL-6 secretion. The expression of ICAM-1 in BeWo cells might be associated to an increase in leukocyte adhesion to the trophoblast barrier, promoting greater inflammation, while the sICAM-1 release could be a protection mechanism activated by trophoblastic cells, in order to regulate the local inflammatory response.

## Background

During *Plasmodium falciparum* infection the infected erythrocytes (IEs) are sequestered in the placental intervillous space (IVS) as a result of the interaction of the parasite’s proteins expressed in the IEs’ surface and chondroitin sulphate A (CSA) on the syncytiotrophoblast’s (ST) surface and IVS [1-3]. The parasite ligand involved on the *P. falciparum* adherent phenomenon is PfEMP-1 (*P. falciparum* erythrocyte membrane protein 1) encoded by members of the *var* multigenic family, composed of about 60 highly variable genes. It has been reported that the PfEMP-1 variant that interacts with the CSA is VAR2CSA codified by the *var2csa* gen [1,2].

Placental infection is characterized by an inflammatory response with monocytes infiltrated in the IVS and cytokines production, such as the tumour necrosis factor (TNF) and gamma interferon (IFNγ) which are immune cellular response mediators and have adverse effects during gestation [3-6]. Infected placentas show increased of inflammatory molecules levels, such as TNF, IL-8, IL-6, and sICAM-1 [3,5,7,8], and chemokines that promote the arrival of immune cells to the placenta, as the macrophage inflammatory protein 1a and 1b (MIP1α and MIP1 β), and monocytes chemoattractant protein (MCP1) has also been reported [8,9].

In the characterization of host-parasite interaction, primary ST and BeWo cellular line have been used; both support CSA-mediated adherence of adhesive parasites [10-13]. Previously it was reported that the ST actively participates in local immune environment modulation in response to the parasite. It has been observed that stimulation of ST with CSA-adherent IEs increases releasing of macrophage migration inhibitory factor (MIF) [14,15] and MIP-1 α [15], activation of signaling cascades (JNK), chemokine gene expression (IL-8 and TGFβ), and stimulates mononuclear chemotactic migration to the placenta [15]. Recently, It has been demonstrated that haemozoin (product of parasite metabolism) also stimulates ST’s immune activation with the ERK1/2 kinase activation and release of chemokines, such as IL-8, MIP1a, and MIP1b, and the soluble form of the ICAM-1 (sICAM-1) receptor, which suggests that this placental barrier responds to malaria parasites [16].

In the placenta, ST is a specialized epithelium barrier that separates the maternal circulation from the villus stroma, regulates foeto-maternal exchange, protects against the passage of infectious agents and participates in recruiting leukocytes into the IVS. The activation of endothelial and epithelial cells by inflammatory and/or infectious agents increases the adhesive properties of these biologic barriers through an increase in the expression of molecules such as ICAM-1 and secretion of chemoattractant molecules [17-22]. The investigation of ST’s contribution to placental malaria pathogenesis and/or protection is essential for the development of therapeutic strategies.

The present study investigated the inflammatory response of BeWo cells (a trophoblast model) mediated by *P. falciparum* CSA-IEs (strains FCB1csa, FCB2csa, FCR3csa and 3D7csa) and TNF in an in vitro cytoadherence model. The results showed that IEs and TNF-stimulated BeWo cells trigger an inflammatory type response, characterized by a surface ICAM-1 increased expression and sICAM-1, IL-8 and IL-6 secretion.

## Methods

### Cellular culture

BeWo human choriocarcinoma cellular line was obtained from American Type Tissue Culture (ATTC, Reference CCL-98) and was cultured following the supplier’s recommendations. The cells were incubated at 37°C in a 5% CO_2_ in air atmosphere in Ham’s F-12 K (Sigma) medium, supplemented with 10% foetal bovine serum and penicillin/streptomycin (Gibco).

This research was approved by the medical research Institute of school of medicine, Antioquia State University.

### *Plasmodiumfalciparum* cultures

*Plasmodium falciparum* 3D7 and FCR3 strains from African origin, FCB1 and FCB2 from Colombia were cultured *in vitro* with A + erythrocytes (haematocrit 1-5%), in RPMI 1640 medium (Sigma) supplemented with 25 mM of HEPES (Sigma); 0,2 mM of hypoxanthine (Sigma); 16 μg/mL of gentamicin (Sigma), 21,6 mM of NaHCO_3_, and 10% of inactivated human serum A+. The cultures were incubated in O_2_ (5%), CO_2_ (5%) and N_2_ (90%) at 37°C [23]. The parasite cultures were routinely tested and found negative for mycoplasma contamination by PCR as described [24].

### Enrichment of mature *Plasmodium falciparum* stages

For cytoadherence and BeWo stimulation experiments, mature parasites (trophozoites and schizonts) were enriched using the gelatin flotation method [25,26]. A volume of IEs was added to ten volumes of 1% type A porcine gelatin (Sigma G2625), the mixture was incubated at 37°C during 40–50 min, allowing the separation of two phases; the supernatant with mature forms was collected and centrifuged at 1,200 rpm for 5 min.

### Selection of CSA-adherent parasites (panning)

To select CSA binding parasites, the FCB1, FCB2, FCR3 and 3D7 *P. falciparum* strains were selected by panning to immobilized CSA receptor. Briefly: culture flasks were coated with a 100 μg/mL CSA (Sigma C9819) at 4°C overnight, the excess of CSA solution was removed and a suspension of mature stages IEs was added for 1 hour at 37°C on a shaker. The non-adhered IEs were removed with three RPMI washings, and added parasites were incubated with medium and fresh erythrocytes for 24 hours, where the schizonts rupture and invasion of new erythrocytes in the parasites adhered to CSA occurs. CSA adherent parasites were transferred to new culture flasks; this procedure was performed consecutively for six times [11,12].

### Cytoadherence assay

BeWo cells growing until 40-50% confluence in Lab-Tek chambers II were incubated with 300 μL of IEs suspension (10% parasitaemia, 1% haematocrit) in adhesion medium (RPMI 1640 supplemented with 0.5% BSA, pH 6.7) for 1 hour at room temperature with continuous agitation (100 rpm). The non-adhered erythrocytes were removed through four immersion washings in adhesion medium. The CSA-IEs adherent were fixated in 2% glutaraldehyde over night and were stained with 1% Giemsa for 45 min. Adhered IEs counting was quantified in 500 nuclei of BeWo cells. Specificity was determined by blocking the interaction with 100 μg/mL of soluble CSA for 30 min before the adhesion assay. The inhibition percentage of adherence was determined as follows: 100 – (number of IEs adhered in presence of soluble CSA/number of IEs adhered in the control)X100 [12].

### Stimulation of BeWo cells with IEs and TNF

The BeWo cells were cultured in six-well plates and kept growing until 90-100% confluence and were stimulated with: CSA-IEs (1 mL, 10% parasitaemia, 3% haematocrit), TNF (10 ng/mL) and the combination IEs + TNF for a period of 16–18 hours. All stimuli were added to the cells in complete F-12 medium. Non-infected erythrocytes (nIEs) were used as control.

### BeWo cells receptor expression

The expression of ICAM-1, CD36 and CSA in the BeWo cells surface was determined using specific mouse monoclonal antibodies, human anti-ICAM-1 (CBL450F, Millipore), human anti-CD36 (MCA722A647, AbDSerotec) and anti-CSA (CS-56 clone, Sigma). Non-specific fluorescence was quantified using an isotype control (MCA928F, AbDSerotec). Cells were recovered using trypsin and were incubated with the antibodies for 40 min at 4°C. The cells were washed in buffer and immediately analyzed using FACS (BeckmanCoulterEpics).

### Chemokine and cytokine expression

The stimulated BeWo cells supernatants were collected and stored at -20°C until use. The levels of cytokine and chemokine release were quantified using standard sandwich ELISA kits for IL-8 (eBiosciencies, 88–8086), IL-6 (eBiosciencies, 88–7066), MCP-1 (eBiosciencies, 88–7399), TNF(eBiosciencies, 88–7346) and soluble ICAM-1 (R&D systems, DY720E), following the product information sheets. The pg/mL concentration per molecule in the supernatants was determined using standard curves.

### Statistical analysis

The variables studied had normal distribution in BeWo cell cultures and were reported as the mean ± standard error (SEM) for experimental groups performed at least four times independently. The changes in variables between treatments were compared by parametric tests using the statistical software GraphadInStat. The T-student test was used for comparison between two treatments (Basal *vs* TNF) and one and two-way ANOVA comparisons between ≥ three treatments (Basal, nIEs, IEs of the four strains) using Bonferroni posttest. Statistical significance was defined with a p-value <0.05.

## Results

### Selection of CSA-adherent parasites and BeWo adherence

**Figure 1:**
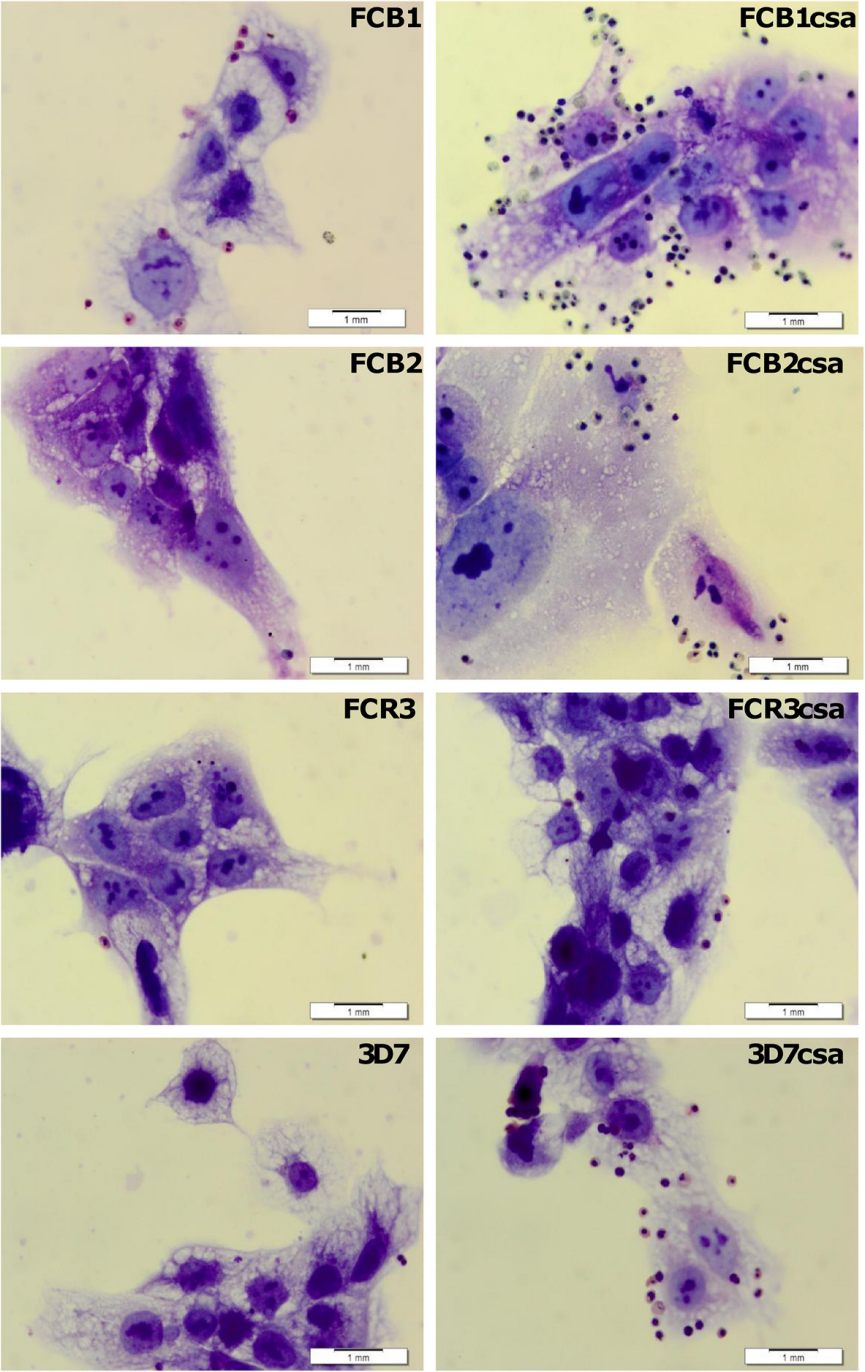
**Adhesion of *Plasmodium falciparum *strains to BeWo cells.** Representative photographs of *P. falciparum* strains adherence BeWo cells at 40-50% confluence (Lab-Tek chambers) were incubated with IEs suspension and stained with Giemsa in order to quantify the number of adhered IEs in 500 nuclei. (1,000× magnification, 1 mm scale).

Before CSA selection, *P. falciparum* strains showed low adherence to BeWo cells, except for FCB1 (Figure 1, Table 1), which presented a greater degree of adherence compared to FCB2, FCR3 and 3D7 strains (p < 0.05). After CSA selection, the strains showed a stable adhesive phenotype, with a significant increase in their adherence (Figure 1, Table 1) compared to their non-panned (P < 0.05). CSA-IEs’ adherence level to the BeWo differs between the selected strains, significant high levels of adherence to FCB1csa and 3D7csa were observed and FCB1csa showed the highest adherence (three to 15 times greater) (P < 0.0001), followed by 3D7csa, FCB2csa, and FCR3csa.

**Table 1:** **Adherence specificity of *Plasmodium falciparum *** **strains of different geographical origin selected to CSA**

**Parasite strain**	**IEs per 500 nuclei**		**% Inhibition**	**p Value**
	**Soluble CSA**			
	**-**	**+**		
**FCB1**	267 ± 35	28 ± 3	88 ± 1,9	< 0,0001
**FCB2**	39 ± 5	15 ± 3	60 ± 7,9	0,0044
**FCR3**	36 ± 7	7 ± 1	72 ± 9,6	0,0018
**3D7**	27 ± 3	12 ± 2	54 ± 7,6	0,0077
**FCB1csa**	1569 ± 133	75 ± 18	95 ± 0,9	< 0,0001
**FCB2csa**	337 ± 33	32 ± 4	91 ± 0,7	< 0,0001
**FCR3csa**	100 ± 12	7 ± 4	94 ± 2,8	< 0,0001
**3D7csa**	595 ± 86	19 ± 8	97 ± 0,8	< 0,0001

Selected strains were incubated with soluble CSA (100 μg/mL) or adhesion medium for 30 min before the cytoadherence assay. The number of adhered IEs to 500 nuclei was quantified, and the inhibition percentage of adherence was estimated. The data represent the mean ± SEM of three independent experiments performed in duplicate (n = 6), t-student for comparison of control *vs* CSA treatment.

IEs pre-incubation with soluble CSA significantly reduced the adherence to BeWo cells in all selected strains, with >90% of inhibition percentages (Table 1), suggesting that the selected strains bind to BeWo cells through a specific interaction with CSA.

### *Plasmodium falciparum* IEs and TNF increase ICAM-1 expression in BeWo cells surface

**Figure 2:**
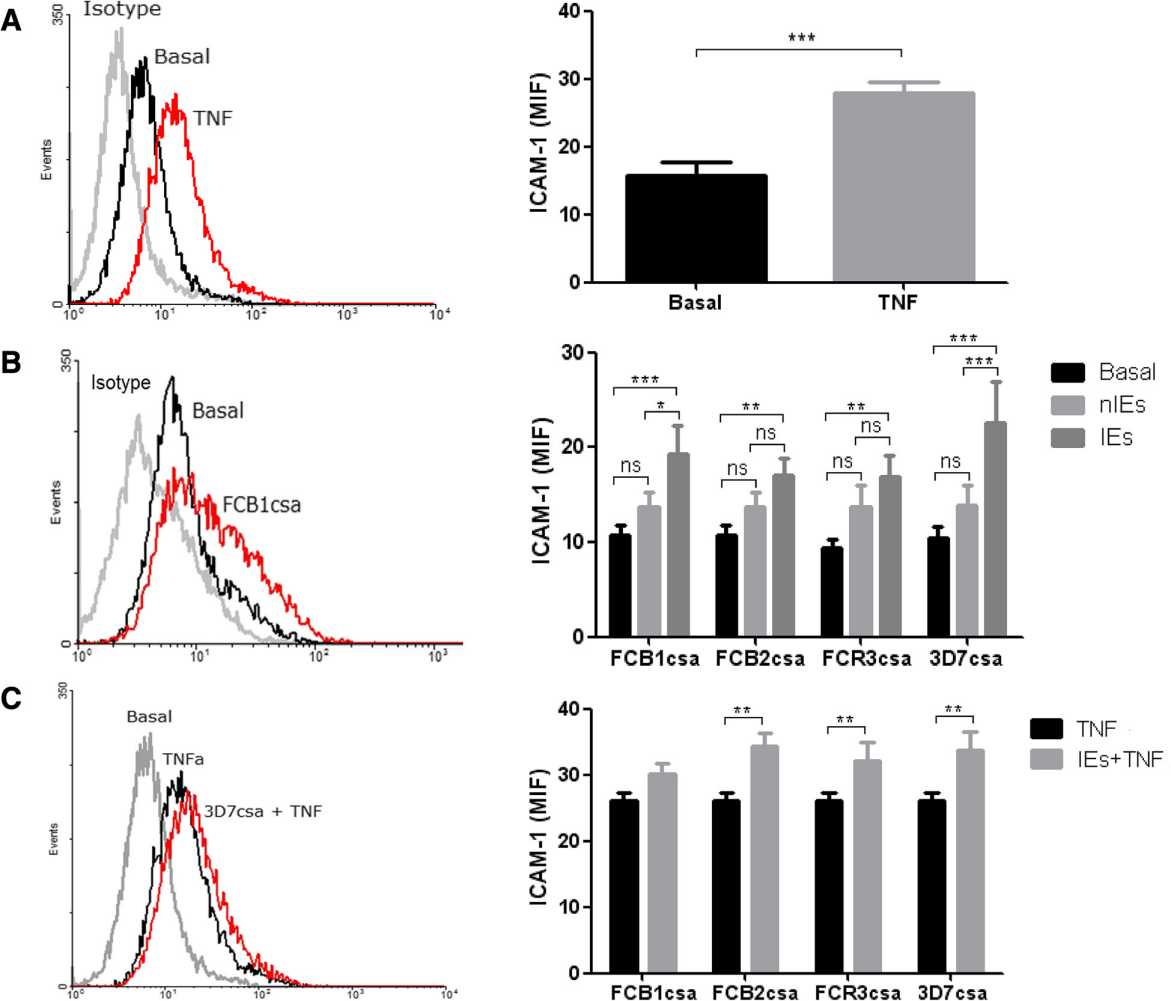
**Effect of TNF and IEs in ICAM-1 expression in BeWo cells’ surface.** Cell cultures were stimulated with 10 ng/ml of TNF **(A)**, IEs **(B)** or the combination of both stimuli **(C)** for 18–20 hours. The cells were incubated with an anti-ICAM1 antibody conjugated with FITC, and were analyzed using flow cytometry. The data represent the mean ± MFI SEM of ≥4 independent measurements. Paired T-student between basal and TNF, two-way ANOVA to compare the effect of IEs of the different strains. *P < 0.05; **P < 0.01; ***P < 0.001.

Stimulation of BeWo cells with TNF (Figure 2A), CSA-IEs (Figure 2B) and the combination of both stimuli (Figure 2C) promoted a significant increase in ICAM-1 levels on the BeWo cells surface compared with the basal levels (p < 0.05). The four strains induced ICAM-1 expression compared to unstimulated cells (P < 0.05), however, the more adherent strains (FCB1csa and 3D7csa)induced a significant increase in ICAM-1 expression on BeWo cell membrane compared to nIEs used as controls (Figure 2B). TNF induced greater increase in ICAM-1 levels and the combination TNF + IEs increased even more this receptor’s expression on the cell surface (Figure 2C).

### Stimuli of BeWo cells with *Plasmodium falciparum* IEs and TNF induce cytokines and receptors release

**Figure 3:**
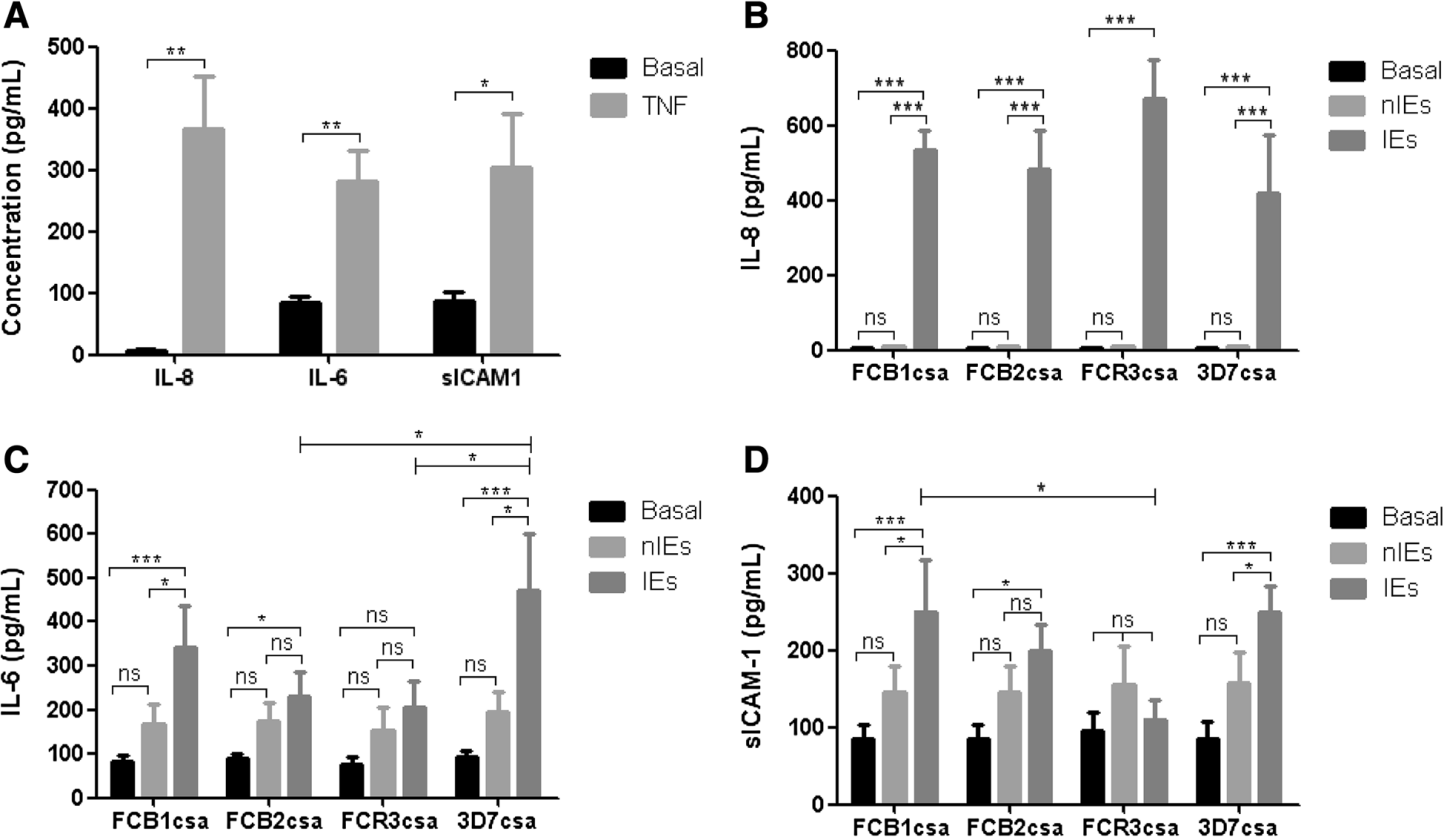
**Exposure of BeWo to TNF and *Plasmodium falciparum-*IEs increases the release of inflammatory molecules.** The production of IL-8, IL-6 and sICAM-1 was determined in BeWo cultures’ supernatants of TNF stimulated and non-stimulated cells by ELISA **(A)** and *P. falciparum*-IEs **(B-D)**. The data represent the mean ± SEM of the concentration in ≥4 separate experiments. Paired T-student between basal and TNF, two-way ANOVA to compare the effect of IEs of the different strains. *P < 0.05; ** P < 0.01; *** P < 0.001.

Non-stimulated BeWo cells constitutively released IL-6 and sICAM-1 and low concentrations of IL-8 were detected in culture’s supernatants (Figure 3A). The addition of TNF increased levels of IL-8, IL-6, and sICAM-1 (Figure 3A). IL-8 levels increased after the incubation with CSA-IEs from the four strains compared to the basal production and nIEs stimulated cells (Figure 3B, p < 0.05). Significant change in IL-6 secretion under stimuli with FCB1csa and 3D7csa strains was found (Figure 3C) and higher increased of IL-6 level was observed for 3D7csa compared to FCB2csa and FCR3csa (strains with lower adherence). The sICAM-1 release increased significantly under treatment with FCB1csa and 3D7csa strains, but not for FCB2csa and FCR3csa (Figure 3D). Production of TNF, MIP-1α and MCP-1 was not detected. TNF + IEs combination did not induce changes in the IL-6 and sICAM-1 in secretion compared to TNF stimuli alone

## Discussion

It is well known that in the placenta, IEs adhere and activate the ST through CSA interaction [12,15]. In this study an *in vitro* placental sequestration model was established using non-syncytialized BeWo trophoblasts cellular line, in order to investigate its response to the parasite binding and to TNF. The findings suggest that BeWo cells responded to CSA-IEs and TNF, promoting a pro-inflammatory environment in the placenta. This suggestion based in the increased mICAM-1 expression and secretion of immune factors, such as IL-8, IL-6 and sICAM-1.

It is important to highlight that the BeWo cell line supports the adherence of IEs via CSA, and it is a useful model for studying parasite-host interactions in placental malaria [10-13]. In agreement with previous reports, it was found that BeWo cells express a receptor profile similar to primary placental ST, characterized by the constitutive expression of surface CSA and ICAM-1, and the absence of CD36 on the cellular surface [11-13]. It is known that *P. falciparum* adheres to ST via CSA, therefore, strains were subjected to CSA panning to make parasites adherent to BeWo cells. After panning the selected parasites adhered to CSA confirming the previous findings showing that parasites with different genetic repertoires can be selected *in vitro* to adhere to CSA [13]. Differences in parasite adherence to BeWo cells were found among the four CSA-strains with the highest adherence observed in FCB1csa strain. The different adhesive profiles observed between strains with different geographical origin and genetic repertoire could be explained by PfEMP-1 molecular and structural characteristics, codified by highly variable genes or by specific VAR2CSA expression levels on the IEs surface.

In the adherence model BeWo cells response to CSA-IEs and TNF stimuli, both implicated in placental malaria pathogenesis [27]. TNF or CSA-IEs of *P. falciparum* increased surface expression of ICAM-1 and appear be related to the adherence ability of IEs because the more adherent lines (FCB1csa and 3D7csa) had a significant effect. Moreover, it was found that the combination of stimuli (resembling the physiological scenario of placental malaria) causes a greater increase in this adhesion molecule levels. ICAM-1 receptor recognizes its ligand (LFA-1 and MAC-1) within immune system cells and promotes their recruitment to inflammation sites [28]. The increase in this receptor’s expression on the ST’s surface is a characteristic of placental inflammatory disorders and promotes immune cells adhesion to ST, augmenting local immune responses.

*In vitro* studies with primary ST cultures suggest that cytokines, such as TNF and/or IFNγ stimulate monocyte-ST adherence through an ICAM-1 increase [18-20], this interaction is important from a pathogenic point of view, since monocytes recruitment can result in ST cell apoptosis and disruption of this barrier in presence of TNF [19]. It has been shown that infectious agents associated to placental inflammatory conditions regulate ICAM-1 in the ST, trophoblasts HIV-1 or cytomegalovirus infection, such as the contact of ST with *Toxoplasma gondii*-infected mononuclear cells, leads to an over-expression of ICAM-1 and promotes an increase of monocytes and lymphocytes adhesion in the ST [20-22]. Supporting these observations, the immunohistochemical analysis of placentas with placentitis by *T. gondii* and *Trypanosoma cruzi* shows an ICAM-1 over-expression in the ST associated to leukocyte infiltrations and trophoblastic barrier loss [29]. Finally, ICAM-1 hyper-expression has been reported on *P. falciparum-*infected placentas [30].

It was observed that upon contact with IEs and haemozoin, the ST releases different cytokines and chemokines [14-16]. *Ex vivo* models support the role of foetal cells in the inflammatory response to malaria infection [4,8]. Release of IL-8, IL-6, sICAM-1, MIP-1α, MCP-1 and TNF from BeWo cells under TNF and IEs-CSA stimulation was investigated and an increase of IL-8, IL-6 and sICAM-1 concentration was found. Less adherent strains (FCR3csa and FCB2csa) induced lower release levels of these cytokines, particularly in IL-6 and sICAM-1, which may suggest that the more adherence of IEs, the more inflammatory reaction. Luchhi *et al.* reported that following IEs interaction, ST increases the mRNA expression of IL-8, the activation of Jun N-terminal kinase 1 (JNK-1) and the secretion of MIF and MIP-1α [15]. Haemozoin stimulated ST cells release molecules, such as IL-8, MIP1a, and MIP1b and sICAM-1 [16]. Reports have been shown that *ex vivo* explants of foetal tissue from malaria-positive placentas secreted significantly amounts of IL-6 [4] and IFN-γ [8] compared to uninfected placentas and its levels has been associated with low birth weight [31]. These findings suggest an inflammatory reaction in the trophoblastic cells, which promotes maternal leukocytes recruitment in the placenta’s surface and then augments inflammatory molecules production.

*Plasmodium falciparum* placental infection is characterized by mononuclear infiltrates in the IVS and local production of inflammatory cytokines and chemokines produced by maternal and foetal cells which increase the arrival and accumulation of mononuclear cells [8,9,32]. TNF increased levels and accumulation of monocytes in the IVS have been associated to low birth weight [3,5,6,32]. Additionally, it has been reported that malarial placentas express higher IL-8 levels, which are associated with placenta leukocytes density and intra-uterine growth delay [9]. Similarly, Fievet *et al.* reported increased IL-6 production in cultures of infected placental villi [4].

An increase in soluble form of ICAM-1 receptor (sICAM-1) was found in supernatant cultures of BeWo cells in response to IEs and TNF. These results suggest that the BeWo cells activation by inflammatory (TNF) or IEs adherent to CSAs results in a mICAM-1 over-expression and in its release from cellular surface to its soluble form or production. It is hypothesized that the ICAM-1 release as a protection mechanism in order to regulate the local inflammatory response [28,33], through to reduce mICAM-1 levels and as consequence the leukocytes adherence, to compete with the membrane-anchored form by the LFA-1 and Mac-1 ligands expressed in leukocytes, which prevents the adherence of these cells to the cellular surface [34] and to contribute to the production of inflammatory molecules [28,35].

Harwell *et al.* found high blood levels of sICAM-1 in *P. falciparum*-infected placentas compared to non-infected placentas’ levels. They also observed a positive association between newborns’ weight to multigravidae women with placental malaria, suggesting that during the infection sICAM-1 released from the placental cells membrane may have an immunoregulatory purpose, in order to reduce load of immune cells in the placenta [36].

Co-culture of late trophozoites and schizonts with non-syncytialized BeWo incubated for up 16–18 hours may have some drawbacks. During parasite maturation schizonts ruptures and release haemozoin, glycosylphosphatidylinositol (GPI) and other parasite-derived products, therefore, it is possible the unspecific stimulation in the BeWo cell model. To determine specific parasite-BeWo activation, short incubation times and unselected-CSA parasites may shed light on parasite-trophoblast interaction/activation. Removing surface adhesive proteins from IEs surface by trypsinization or blocking adherence with soluble CSA should help to establish a relationship among cytoadherence and cell activation. Non-syncytialized BeWo cells showed cell response and adhesion patterns, however as BeWo cells were not syncytialized then it should be considered for further adherence and cytokine expression experiments.

## Conclusion

This study supports previous observations of the immunological response of ST to the infection and particularly describes that CSA-adherent IEs and TNF activate immunologic responses in the BeWo cells, characterized by an increase in the mICAM-1 expression, sICAM-1 release, and secretion of pro-inflammatory cytokines, such as IL-8 and IL-6. Trophoblastic activation contributes to the increase in local levels of inflammatory molecules that play a central role in the migration and accumulation of leukocytes in the placental IVS, and has an important role in pathogenesis and/or local protection. Deeper comprehension of the parasite-host placental interaction might improve intervention strategies.

### Consent

This research was performed with lab strains of Plasmodium falciparum (FCB1, FCB2, 3D7 and FCR3) and does not include human samples.

## Competing interests

The authors declare that they have no competing interests.

## Authors’ contributions

AMV carried out laboratory work, design of the study, performed statistical analysis and drafted the manuscript, SB and CS conceived, coordinated the study and participated in the data analyses. All authors read and approved the final manuscript.
